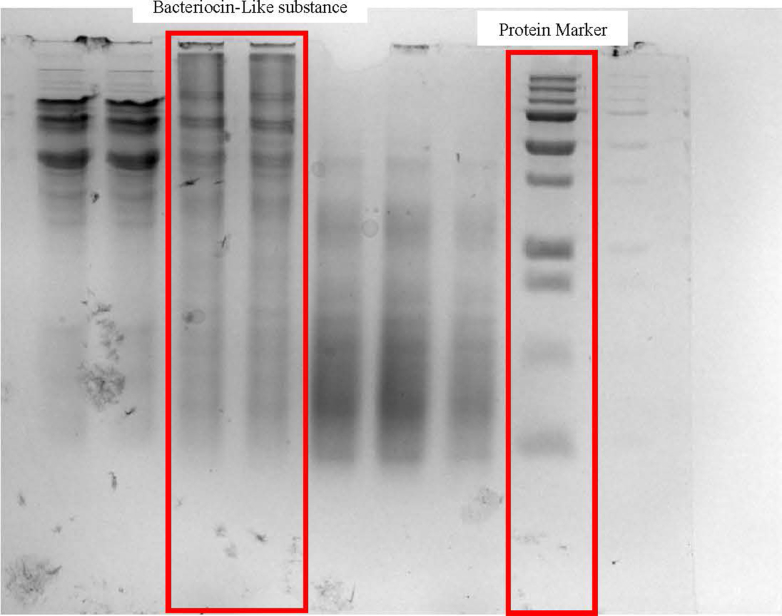# Investigating the production and synergistic antibacterial activity of bacteriocin-like substance from *Brevibacillus laterosporus* SA-14 (TISTR 2453) for enhanced wound healing

**DOI:** 10.1016/j.heliyon.2025.e42510

**Published:** 2025-02-06

**Authors:** Husna Madoromae, Apichart Atipairin, Malatee Tayeh, Monthon Lertcanawanichakul

**Affiliations:** a School of Pharmacy, Walailak University, Nakhon Si Thammarat, 80161, Thailand; b Drug and Cosmetic Excellence Center, Walailak University, Nakhon Si Thammarat, 80161, Thailand; c School of Allied Health Sciences, Walailak University, Nakhon Si Thammarat, 80161, Thailand; d Food Technology and Innovation Research Center of Excellence, Walailak University, Nakhon Si Thammarat, 80161, Thailand

**Keywords:** Anti-MRSA, Bacteriocin-like substance, *Brevibacillus laterosporus*, Cytotoxicity, Synergistic effect, Wound healing

## Abstract

The rise in antimicrobial-resistant (AMR) bacteria, especially Methicillin-resistant *Staphylococcus aureus* (MRSA), is a global health concern. Bacteriocins are promising antibiotic alternatives. This study aimed to enhance the production of bacteriocin-like substances (BLS) from *Brevibacillus laterosporus* SA-14 (TISTR 2453) by optimizing nutrients, evaluating antibacterial activity, assessing synergy with vancomycin, and testing the cytotoxicity and wound healing effects on human keratinocytes. The results showed that when the SA-14 strain was cultured in half-formula Luria-Bertani broth (LB/2) with added carbon sources (glucose, sucrose, and lactose), all cultures reached the late log phase at 24 h, and antibacterial activity was exhibited against various MRSA strains after 48 h, except for the LB/2 supplemented with glucose, likely due to carbon catabolite repression. However, the addition of nitrogen sources, including skim milk, peptone, and beef extract resulted in high antibacterial activity at 48 h, with skim milk being the most effective for BLS production. The BLS was precipitated with 80 % ammonium sulfate, achieving a 38.09 % yield and a protein concentration of 6.97 ± 1.12 mg/mL. The SDS-PAGE analysis revealed five bands of proteins with molecular weights of 25–250 kDa. The minimum inhibitory concentration of BLS ranged from 0.44 to 0.87 mg/mL, with an minimum bactericidal concentration) of 0.87 mg/mL for all MRSA strains. A synergistic effect with vancomycin was observed at 0.22 mg/mL BLS and 1 μg/mL vancomycin, with an fractional inhibitory concentration index of 1.00, indicating an additive effect. At a concentration of 0.22 mg/mL, BLS was non-cytotoxic to HaCaT cells and promoted complete wound healing after 48 h. Therefore, BLS produced by the SA-14 strain is suitable for controlling AMR, especially MRSA, and has the potential for application in wound dressings in the future.

## 1 Introduction

Antimicrobial resistance (AMR) poses a serious threat to global health and food security. AMR can result in longer hospital stays, higher medical costs, and higher mortality rates. The World Health Organization (WHO) has identified AMR as one of the top ten global public health threats. It is estimated that AMR infections were responsible for 1.27 million global deaths in 2019 and contributed to 4.95 million deaths worldwide [1]; the mortality rate is predicted to reach 10 million people by 2050 [2]. The World Bank reported that AMR infections will cause an increase in healthcare costs by 1 trillion US$ by 2050, and US$ 1 trillion to US$ 3.4 trillion in gross domestic product (GDP) losses per year by 2030 [3]. The incidence of AMR infections in Thailand is reported to be 87,751 drug-resistant illnesses and 38,481 deaths per year [4]. The six leading pathogens responsible for deaths associated with resistance, including *Escherichia coli*, *Staphylococcus aureus*, *Klebsiella pneumoniae*, *Streptococcus pneumoniae*, *Acinetobacter baumannii*, and *Pseudomonas aeruginosa* were responsible for 929,000 deaths attributable to AMR and 3.57 million deaths associated with AMR in 2019. One pathogen-drug combination, methicillin-resistant *S. aureus* (MRSA), caused more than 100,000 deaths attributable to AMR in 2019 [1], and the median reported rate of 35 % for MRSA infections in 76 countries is a major concern [1].

Methicillin-resistant *S. aureus* (MRSA) is a bacterial resistance that is a global concern. Many studies have reported that people infected with MRSA are 64 % more likely to die than those with drug-sensitive infections [1]. Antibiotics are currently ineffective against MRSA, including ciprofloxacin, gentamicin, clindamycin, moxifloxacin, and chloramphenicol, except vancomycin, daptomycin, and linezolid [5,6]. Moreover, vancomycin is the major antibiotic that has long been considered the most effective for treating MRSA infections [7]; however, the adverse effects of vancomycin, including neutropenia, nephrotoxicity, diarrhea, and nausea, are generally well tolerated. Currently, there is an increasing focus on studying natural substances that can reduce drug use. Bioactive compounds (BCs) are among the most extensively studied compounds because of their potential to combat MRSA infections [[8], [9], [10]] and their safety for medical use. In particular, bacteriocins produced by bacteria, as a type of BCs, have shown significant promise [8,[11], [12], [13]].

Bacteriocins and bacteriocin-like substances (BLS) are secondary metabolites. These peptides are naturally synthesized by both Gram-positive bacteria (GPB) and Gram-negative bacteria (GNB) to help them survive in changing environments by providing protection against competing microorganisms. It contains antimicrobial agents, particularly antimicrobial peptides (AMPs) and BLS, such as angicin produced by *Streptococcus anginosus* showed high activity against closely related *streptococci*, *listeria* and vancomycin resistant *enterococci* [14], and bacin A1, A2, A3, and A4, produced by *Bacillus* sp. TL12 which show potent bactericidal effect against *S. aureus* and MRSA [15]. In addition, *Brevibacillus* spp. are extensively scattered throughout nature and can be found in the soil, water, and air. They are also recognized to be a rich source of several antibacterial peptides [8,11,13]. Several bioactive compounds produced by *Brevibacillus* spp. have been reported, including *Brevibacillus* sp. SPR-20 produces an anti-MRSA peptide against *S. aureus* and MRSA [16]. Iturin-like lipopeptides produced by *Brevibacillus* spp. strain GI9 have demonstrated a broad spectrum of activities that inhibit the growth of various fungi [17]. Similarly, bogorol-like lipopeptides produced by the *Brevibacillus* spp. strain SKDU10 showed broad-spectrum antimicrobial activity [17]. Furthermore, the SKDU10 strain produced laterosporulin 10, which is effective against *S. aureus* and *Mycobacterium tuberculosis* (Mtb H37Rv) [18]. Interestingly, the BLS produced by *Brev. laterosporus* also show broad-spectrum antimicrobial activity [8,11] and are effective against drug-resistant bacteria [8,19] such as BLS produced by *Brev. laterosporus* G4, which kills nematodes in 12 h [20] and tauramamide, a BLS produced by *Brev. laterosporus* PNG276 exerts broad-spectrum antimicrobial effects [21].

In previous studies, *Brev. laterosporus* SA-14 (TISTR 2453), a Gram-positive bacillus with aerobic spore formation, was isolated from air samples at Walailak University, Thailand, and found to produce bacteriocins or BLS [22]. The SA-14 strain can produce BLS in Luria-Bertani (LB) broth on the first day of culture [19] and shows a higher amount of BLS on days 8–10 of culture [13]. BLS produced by the SA-14 strain can inhibit the growth of GPB and GNB, especially *S. aureus* and MRSA [13,22], and is more potent than vancomycin and oxacillin [23]. Moreover, it is more stable in acidic and basic environments, does not exert cytotoxic effects on human white blood cells (WBC), and can stimulate WBC proliferation; however, hemolytic activity on red blood cells (RBC) has been observed [24]. Currently, the industrial applications of BLS are interesting but challenging for researchers. Bacterial production must be enhanced to increase the volume and quality of the products. Additionally, antimicrobial peptides (AMPs) should meet specific conditions for practical applications, including the capacity for high-purity and high-quality large-scale production. There are many factors involved, both physical and chemical factors that can affect BLS production. Researchers are also working to demonstrate the safety of BLS for use in humans, animals, and the environment and to explore its applications in pharmaceutical, medical, agricultural, and other fields. In addition, the wound-healing properties of BLS produced by *Brevibacillus* spp. have not been studied extensively. This study aimed to enhance the production of bacteriocin-like substances (BLS) from *Brevibacillus laterosporus* SA-14 (TISTR 2453) by optimizing nutrients, evaluating antibacterial activity, assessing synergy with vancomycin, and testing the cytotoxicity and wound healing effects on human keratinocytes.

## 2 Materials and methods

The assignment of a biosafety level to a particular work process was made through a protocol-driven risk assessment. This research was processed under biosafety performance in clearance no. WU-IBC-66-004 from the Institute Biosafety Committee of Walailak University.

### 2.1 Bacterial strains and culture condition

The *Brev. laterosporus* SA-14 (TISTR 2453) was isolated from an air sample collected at Walailak University, Nakhon Si Thammarat, Thailand [22]. The SA-14 strain was cultured on half formula of Luria-Bertani (LB/2) (6.25 g of LB [Himedia, India] and 7.50 g of agar [Himedia, India]) incubated at 37 °C for 48 h.

The indicator organisms *Staphylococcus aureus* TISTR 517, clinically isolated from methicillin-resistant *Staphylococcus aureus* 142, 1429, 1096, 2499, and *Escherichia coli* TISTR 887, were provided by the Medical Technology Laboratory, School of Allied Health Sciences, Walailak University, Nakhon Si Thammarat, Thailand. All indicator organisms were cultured on Mueller-Hinton agar (MHA) (Himedia, India) and incubated at 37 °C for 24 h.

### 2.2 Optimization factors

A single colony of SA-14, approximately 4 mm in size, was inoculated into a 25 × 150 mm screw cap test tube containing 10 mL of LB/2 broth medium and used rattler platting beads diameter 4.5 mm (Zymo Research, USA) in a 1:1 media ratio for prepared starter and incubated at 37 °C, 150 rpm for 24 h. Then, adjust the turbidity of starter to McFarland No. 0.5 and 1 % of stater was inoculated to 200 mL of LB/2 broth supplemented with 2 mg/mL of different carbon sources including glucose (Pronadisa, Spain), lactose (Ajex FineChem, Australia), sucrose (Ajex FineChem, Australia) and 2 mg/mL of different nitrogen sources including beef extract (Himedia, India), skim milk (Himedia, India), peptone (Himedia, India) and incubated at 37 °C, 150 rpm for 96 h.

The growth curve was determined at 0, 2, 4, 6, 8, 12, 18, 24, 48, 72, and 96 h by measuring the turbidity at OD 600 nm. The suspension was centrifuged at 10,000 rpm, 30 min, 4 °C to collect the cell-free supernatant (CFS) for determining the antimicrobial activity and protein concentration.

### 2.3 Production of bacteriocin-like substance (BLS)

The starter of the SA-14 strain was prepared, the turbidity of the starter culture was adjusted to McFarland No. 0.5, and 1 % was inoculated into 200 mL of LB/2 broth medium containing rattler plating beads with a diameter of 4.5 mm (Zymo Research, USA) in a 1:1 ratio. The culture was incubated at 37 °C and 150 rpm for 48 h. After incubation, the culture broth was harvested and the cell sediment was separated by centrifugation at 4 °C and 10,000 rpm for 30 min. The supernatant was kept at 4 °C for further testing.

### 2.4 Determination of antibacterial activity using the cross-streak assay

The antimicrobial activity was determined using the cross-streak method, with modifications from a previous study [25]. Two to four colonies of the SA-14 strain were streaked with a sterile loop in the center of the agar plates and incubated at 37 °C for 4 days. The indicator microorganism was prepared, the turbidity for McFarland No. 0.5 was determined using sterile normal saline solution, the microorganism solution was streaked onto a plate at a 90-degree angle and incubated at 37 °C for 18–24 h, and the result was read.

### 2.5 Determination of antibacterial activity using the agar well diffusion assay

Prepared Mueller-Hinton Agar (MHA) was used as the medium. All indicator microorganisms were prepared from fresh colonies, and their turbidity was adjusted to McFarland standard No. 0.5 using sterile normal saline solution. The microorganisms were then swabbed onto MHA. A hole was drilled using a 1000-μL sterile tip, which was discarded afterward using the sterile technique. A 100-μL sample of CFS and BLS was loaded into the holes, and the plates were allowed to rest at room temperature until the substances were absorbed into the agar. Culture medium without cells served as a negative control. The plates were then incubated at 37 °C for 18–24 h, and the clear zones were observed and recorded.

### 2.6 Minimum inhibitory concentration and minimum bactericidal concentration

The MIC and MBC of BLS produced by the SA-14 strain and vancomycin were determined using a 96-well plate. Bacterial indicators were streaked onto Mueller-Hinton agar and incubated at 37 °C for 18–24 h. The turbidity of the bacterial indicators was adjusted to 10^6^ CFU/mL using sterile normal saline. A stock solution of vancomycin (1 mg/mL) was diluted to 128 μg/mL. Fifty microliters of 4x MHB was added to each well, followed by 50 μL of the 128 μg/mL vancomycin solution to wells 1A–1C with a 2-fold serial dilution. Similarly, 50 μL of the BLS was added to wells 1F–1H, followed by a 2-fold serial dilution. Sterile normal saline and sterile MHB served as negative controls, and 64 μg/mL vancomycin as the positive control. Fifty microliters of each bacterial indicator suspension was added to the respective wells and incubated at 37 °C for 18–24 h. Resazurin was added to assess cell viability, and the samples were incubated at 37 °C for 30–45 min. The breakpoint for MIC in this study was defined as the treatment concentration that caused a 50 % reduction in metabolic activity, indicated by the color intensity of resazurin, compared to the untreated control. The MHA plates were then streaked with culture broth to confirm the MIC and MBC, and the results were recorded.

### 2.7 The semi-purification of BLS produced by SA-14 strain

A volume of 3000 mL of CFS was transferred to a beaker with a magnetic stirrer (Pyrex, Germany). The 80 % ammonium sulfate ((NH_4_)_2_SO_4_) (Lab-Scan, Thailand) was slowly added to CFS and incubated overnight at 4 °C. The precipitate was centrifuged at 10,000 rpm for 30 min, the supernatant was discarded, and the precipitated protein was dissolved in 10 mL of phosphate-buffered saline (PBS) (Merck, Germany) until completely dissolved. Ammonium sulfate was eliminated using a dialysis bag (Merck, Germany) with a molecular weight cut-off of 3 kDa, soaked in PBS (Merck, Germany) every hour for 4 h, and incubated at room temperature overnight. Centrifuged at 10,000 rpm at 4 °C for 30 min to get the BLS for further testing.

$$\%\;\text{Yield}=\frac{\text{Total}\;\text{protein}\;\text{before}\;\text{precipitation}}{\text{Total}\;\text{protein}\;\text{after}\;\text{precipitation}}\;x\;100$$

### 2.8 Protein determination

The concentration of the protein standard was determined according to the manufacturer's instructions (Thermo Fisher Scientific, USA). Briefly, CFS and BLS were diluted 2-fold, Bradford reagent was added to each tube, incubated for 10 min at room temperature, and the concentration was determined using a spectrophotometer (Thermo Spectronic GENESYS 20, USA) at a wavelength of 595 nm (measured within 1 h). A standard curve illustrating the correlation between absorbance and standard solution concentration was prepared using bovine serum albumin (BSA) standard protein (Merck, Germany) at concentrations of 0, 0.2, 0.4, 0.6, 0.8, and 1.0 mg/mL.

### 2.9 Determination of molecular weight

The molecular weight of BLS was determined using sodium dodecyl sulfate-polyacrylamide gel electrophoresis (SDS-PAGE), as previously described [26]. SDS-PAGE (Merck, Darmstadt, Germany) was performed according to the manufacturer's instructions. BLS was prepared by mixing with 6x loading dye (Bio-Rad). The samples were boiled at 95 °C for 5 min, then 10 μL of this and low molecular weight protein markers (Bio-Rad, USA) were loaded into 15 % SDS-PAGE (Merck, Germany), after electrophoresis, the gel pads were stained with 0.1 % Coomassie blue in 40 % ethanol and 10 % acetic acid (Merck, Germany) using a rotator for 30–60 min, or until the resulting protein bands were visible. The gel was shaken thoroughly and washed with destaining solution for 2–3 h or until clear.

### 2.10 Synergistic effect by using checkerboard assay

All bacterial indicators were streaked on MHA and incubated at 37 °C for 18–24 h. The bacterial indicators were then adjusted to a concentration of 10^6^ CFU/mL using sterile normal saline. Vancomycin and BLS were prepared at 8 times the MIC. A stock solution of vancomycin was prepared at a concentration of 1 mg/mL and then diluted to 16 μg/mL. Fifty microliters of 4x MHB was added to each well. Subsequently, 50 μL of the vancomycin solution at a concentration of 16 μg/mL was added to each well in row A, covering columns 1 to 11. A two-fold serial dilution was then performed by transferring 50 μL from row A to row B, and this process was continued from row B to row G. The remaining 50 μL from row G was discarded. In parallel, 50 μL of the BLS solution was added to each well in column 12, followed by a two-fold serial dilution from column 12 to column 2, with the remaining 50 μL from column 2 being discarded. Sterile normal saline and sterile MHB served as negative controls, while 32 μg/mL vancomycin was used as the positive control. Fifty microliters of each bacterial indicator suspension were added to the respective wells, as labeled, and incubated at 37 °C for 18–24 h. Resazurin was then added to the culture broth and incubated at 37 °C for 30–45 min. The results were recorded, and the fractional inhibitory concentration index (FICI) was calculated using the formula as described by Ref. [27].

$$\text{FICI}=\frac{A}{\text{MIC}\left(A\right)}+\frac{B}{\text{MIC}\left(B\right)}$$

A = The MIC value of vancomycin in combination with BLS.

B = The MIC value of BLS in combination with vancomycin.

MIC(A) = The MIC of vancomycin alone.

MIC(B) = The MIC of BLS alone.

### 2.11 In vitro cytotoxicity on HacaT cells

BLS cytotoxicity was investigated using keratinocytes (HaCaT cells). The cell culture was prepared by growing the cells in Dulbecco's Modified Eagle Medium (DMEM) supplemented with 10 % fetal bovine serum and 1 % antibiotic-antimycotic (anti-anti), and incubated at 37 °C with 5 % CO_2_ for 48 h. HaCaT cells 100 μL were seeded at a density of 1 × 10^4^ cells per well and incubated at 37 °C with 5 % CO_2_ for 24 h. CFS and BLS were prepared by making 2-fold dilutions in DMEM +10 % fetal bovine serum (FBS). The culture medium was discarded, and 100 μL of each concentration of CFS and BLS was added to the wells, followed by incubation under the same conditions for 24 h. The treated cells medium was then discarded, and 100 μL of 0.5 mg/mL 3-(4,5-dimethylthiazol-2-yl)-2,5-diphenyl tetrazolium bromide (MTT) solution was added and incubated under the same conditions for 3–4 h. After the incubation period, the culture medium was removed, and the dark blue formazan crystals were dissolved in 100 μL of dimethyl sulfoxide (DMSO). Cell viability was determined at 570 nm using a spectrophotometer. The percentage of viable cells was calculated using the following formula as described by Ref. [28].

$$\text{Relative}\;\text{cell}\;\text{viability}\left(\%\right)=\frac{\text{OD}\;\text{of}\;\text{sample}}{\text{OD}\;\text{of}\;\text{NG}}\;x\;100$$

OD of sample = The optical density of the test material.

OD of NG = The optical density of the negative control.

### 2.12 Wound healing scratch assay

A wound-healing scratch assay was performed to evaluate wound closure in HaCaT keratinocytes. The cells were seeded in a 6-well plate at a density of 6.8 × 10^4^ cells/mL. Two milliliters of cell suspension in DMEM with 10 % FBS were added to each well and incubated at 37 °C with 5 % CO_2_ for 24 h. The cell cultures were starved in serum-free DMEM and incubated under the same conditions for another 24 h. A sterile 200 μL tip was used to make a linear scratch in the monolayer, and the cells were washed carefully with PBS 2–3 times. BLS was diluted to 6.5 %, 3.13 %, and 1.5 % in DMEM without serum, and 2 mL of each concentration was added to the respective test wells. The positive control was 10 % FBS, whereas the negative control was DMEM without serum and PBS. The test plates were incubated under the same conditions. To assess wound closure, four images of each well were captured using a Leica DMLS microscope at 10 × magnification 0, 24, 36, and 48 h after treatment. The data were analyzed using the ImageJ software. Percentage wound closure was calculated using the following formula:

$$\%\;\text{Wound}\;\text{closure}=\frac{{\text{Gap}\;\text{distance}}_{t0}-{\text{Gap}\;\text{distance}}_{t24}}{{\text{Gap}\;\text{distance}}_{t0}}\times 100$$

### 2.13 Statistical analysis

The statistical analysis was carried out using GraphPad Prism version 10. Data obtained from the assays performed in triplicate were analyzed for antimicrobial activity, protein concentration, cytotoxicity, and percentage of wound closure. The results were expressed as mean ± standard deviation (SD). Comparisons of activity before and after precipitation were analyzed using two-way ANOVA, with a p-value of <0.05 considered significant.

## 3 Results and discussions

### 3.1 Screening of the antimicrobial activity of *Brevibacillus laterosporus* SA-14 (TISTR 2453) strain using the cross-streak method

**Fig. 1:**
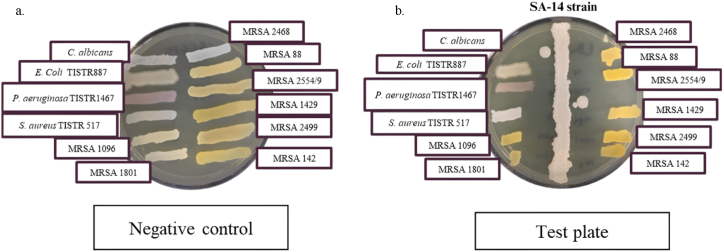
Antibacterial activity of SA-14 strain by using cross streak method, (a.) the negative control plate showed full line growth of all indicator organisms across the entire plate, (b.) the test plate showed a declining pattern of growth for all indicator organisms.

This study used the cross-streak method to screen the antibacterial activity of the SA-14 strain. The results revealed that in the test plate (Fig. 1b), growth along the indicator line was shorter than that of the control plates (Fig. 1a), which exhibited full-line growth. This suggests that the SA-14 strain produces substances with antimicrobial activity, particularly anti-MRSA activity. Consistent with previous studies, the SA-14 strain demonstrated efficacy against both GPB and GNB, particularly showing significant effects against MRSA [13,22]. Additionally, *Brev. halotolerans* 7WMA2 exhibits a broad spectrum of antifungal activity against several fungi, including *Alternaria alternata*, *Cladosporium* sp., and *Candida albicans* [29]. Moreover, in the cross-streak method used for screening antibacterial activity from soil, 94 isolates were found to be active against Gram-positive *S. aureus* TISTR 517 and 67 isolates were active against Gram-negative *E. coli* TISTR 887 indicator strains [30]. Furthermore, the cross-streak method is particularly suitable for initial screening of antimicrobial activity because of its simplicity, cost-effectiveness, and ability to quickly identify potential antimicrobial agents from a large number of isolates.

### 3.2 Selection of basal medium

**Fig. 2:**
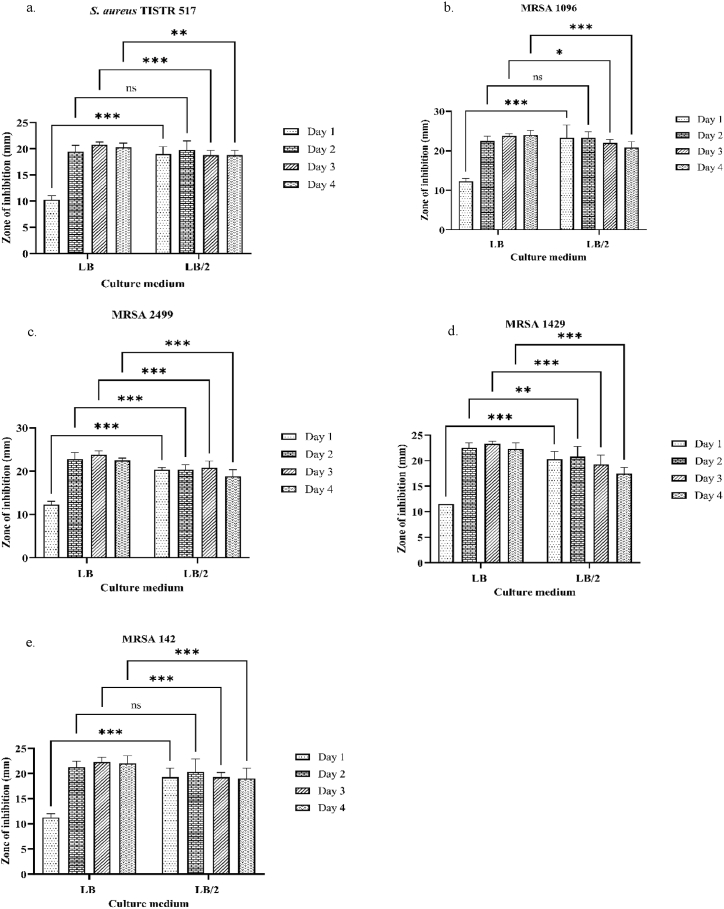
Antibacterial activity of SA-14 strain in LB compared with that in LB/2, ∗The antibacterial activity in LB and LB/2 is significant, ns = The antibacterial activity in LB and LB/2 is not significant, (a.) antibacterial activity against *S. aureus* TISTR 517, (b.) antibacterial activity against MRSA 1096, (c.) antibacterial activity against MRSA 2499, (d.) antibacterial activity against MRSA 1429, (e.) antibacterial activity against MRSA 142.

Luria-Bertani broth (LB) is a nutrient-rich medium commonly used for bacterial cultivation. They reported that *Brev. laterosporus* strains BGSP7, BGSP9, BGSP11, and BGSP12 cultured in LB broth produced BLS against *S. aureus* ATCC25923, *Listeria monocytogenes* ATCC19111*, Klebsiella pneumoniae* Ni9, and *Pseudomonas aeruginosa* MMA83 within the first day of cultivation and showed high activity by the third day [8]. Similarly, antimicrobial activity was exhibited by the SA-14 strain cultured in LB broth after 8–10 days of cultivation [13]. Additionally, there have been indications that antimicrobial activity can also be observed as early as the first day of cultivation [19]. In this study, the basal medium was selected by inoculating the SA-14 strain into LB broth and comparing its antibacterial activity with that in LB/2. The results demonstrated that the LB/2 culture broth exhibited significantly superior antibacterial activity against all bacterial indicators on the first day of cultivation (Fig. 2). The highest antibacterial activity was observed on the second day of cultivation, significantly against MRSA 2499 and MRSA 142 (Fig. 2c and d), but not against *S. aureus* TISTR 517, MRSA 1096, or MRSA 1429 (Fig. 2 a–b, e), compared to LB. The SA-14 strain produces BLS in the late log phase of bacterial growth, during which bacteria utilize available nutrients, leading to starvation and the production of BLS for survival [31]. It has been reported that a lack of oxygen and glucose causes *K. pneumoniae* to rapidly enter the stationary phase, during which it produces ampicillin and ciprofloxacin for self-protection [32]. In the present study, the reduced nutrient concentrations in LB/2 caused the SA-14 strain to utilize nutrients more quickly, leading to faster starvation and stress. This accelerated the transition to the late log phase, resulting in increased production of antibacterial substances compared with that in full LB medium within the same time period. This finding aligns with previous research showing that the GPB *Streptomyces* KB1 (TISTR 2304) cultured in both LB and LB/2 entered the late log phase simultaneously and exhibited no significant antibacterial activity within the same period [33]. Additionally, the full formula of LB, rich in essential nutrients, supports primary metabolism for cell growth [34,35], causing bacteria to enter the late log phase. Furthermore, the antibacterial properties of LB/2 were enhanced to be comparable to those of the full LB formula, while offering cost savings. Therefore, the SA-14 strain cultured in LB/2 for 2 days was a suitable basal medium for this study.

### 3.3 Effect of carbon sources

**Fig. 3:**
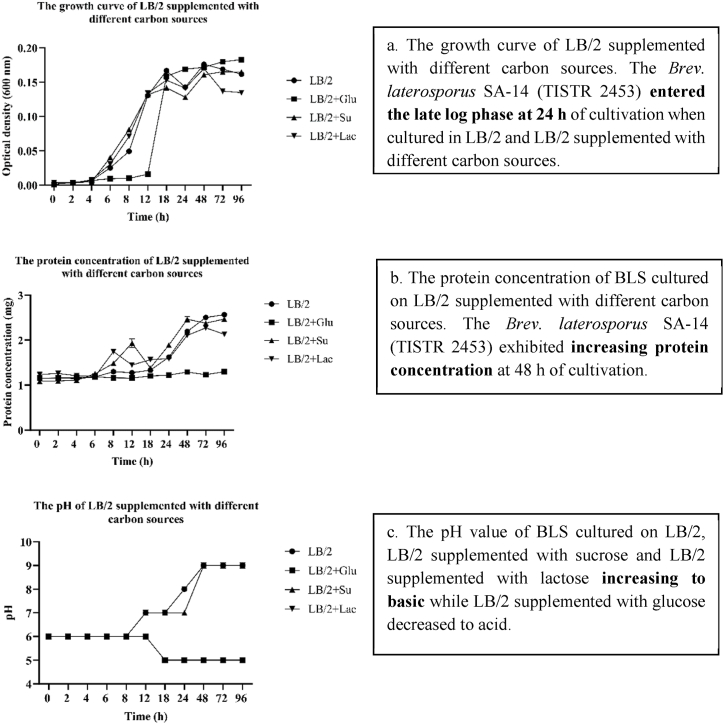
The bacteriocin-like substances (BLS) produced by SA-14 strain culture on LB/2 supplement with different carbon sources were improved, (a.) growth curve of SA-14 strain cultured on LB/2 supplemented with different carbon sources (glucose, sucrose, and lactose), (b.) protein concentration of BLS in different carbon sources (glucose, sucrose, and lactose), (c.) pH value of BLS in different carbon sources.

Carbon sources are important for supporting bacterial growth, as they serve as primary energy and biomass building blocks. Bacteria utilize carbon sources in various metabolic processes to produce energy (ATP) and synthesize essential cellular components such as proteins, nucleic acids, lipids, and carbohydrates [36]. Different bacteria have varying preferences for, and abilities to metabolize, different carbon sources, which can influence their growth rate, biomass yield, and overall metabolic activity [37]. In this study, the basal medium (LB/2) was supplemented with different carbon sources, and the results showed that LB/2 and LB/2 supplemented with different carbon sources entered the late log phase after 24 h of cultivation (Fig. 3 a). However, only a minimal amount of the substance was detected, which resulted in no observable antibacterial effects. This finding corresponds to the low protein concentration observed during the same period, which increased after 48 h (Fig. 3 b) and the pH value was basic (Fig. 3 c). The antibacterial activity of carbon sources was significant at *p* < 0.05 at 48 h of cultivation in LB/2, LB/2 supplemented with sucrose, and LB/2 supplemented with lactose (Table 1). LB/2 medium supplemented with sucrose exhibited significantly decreased anti-MRSA activity against all MRSA strains, whereas LB/2 supplemented with lactose showed notably better activity against *S. aureus* TISTR 517 but was less effective against MRSA strains 1096, 2499, and 142 (Table 1). In addition, the LB/2 cells supplemented with glucose showed no antibacterial activity (Table 1). Consistent with previous studies, where exhibited that glucose strongly represses the growth of *Bacillus subtilis* more than malate. This is because glucose preferentially activates regulatory pathways that inhibit the utilization of alternative carbon sources, such as malate, which serves as a more efficient energy source. This leads to increased levels of adenosine triphosphate (ATP) and fructose-1,6-bisphosphate, which activate HPr kinase and repress genes associated with malate uptake and metabolism. As a result, glucose not only affects the growth rate of *B. subtilis* but also its metabolic flexibility and adaptability to varying nutrient conditions [38,39]. LB/2 supplemented with different carbon sources significantly decreases antibacterial activity because *Bacillus* spp. such as *B. subtilis* produce α-amylase (hydrolytic enzymes) to digest polysaccharides into monosaccharides, which cells can use as an energy source during the exponential growth phase and stationary phase [40]. Supplementation of LB/2 with different carbon sources is not suitable for improving BLS production in the SA-14 strain, because bacteria use glucose/sugar transport proteins on their cell membranes to take up glucose and other monosaccharides through the Phosphotransferase System (PTS) and ATP-Binding Cassette (ABC) transporter pathways [41]. High concentrations of carbon sources can repress the production of BLS or secondary metabolites via a mechanism known as carbon catabolite repression [42,43]. When bacteria have rich carbon sources, they repress the synthesis of other catabolite enzymes and secondary metabolites by controlling cAMP (cyclic AMP) and CRP (cAMP receptor protein). When glucose concentrations are high, levels of cAMP are decreased, thus playing an important role in stimulating the expression of genes involved in the metabolism of other nutrients and the creation of secondary metabolites. cAMP and CRP work together to form a complex (cAMP-CRP complex) that stimulates the expression of these genes [41,44]. When cAMP levels are reduced, the formation of the cAMP-CRP complex decreases, leading to reduced expression of genes involved in the metabolism of other nutrients and the production of secondary metabolites [41,44]. This causes bacteria to focus on glucose metabolism to produce ATP and primary metabolites instead, which is consistent with previous research findings. When LB/2 was supplemented with glucose, the antibacterial activity disappeared, and the pH value decreased to acidic levels. This is likely due to acid production during fermentation by the SA-14 strain, which produces acids such as lactic acid.

**Table 1:** Antibacterial activity of SA-14 strain compared to the basal medium (LB/2) with and without supplementation of different carbon sources.

Zone of inhibition (mm ± SD)					
Organisms	NC	0–24 h	48 h	72 h	96 h
**LB/2**					
*S. aureus* TISTR 517	0.00 ± 0.00	0.00 ± 0.00	19.00 ± 1.80	20.00 ± 1.00	19.56 ± 1.33
MRSA 1096	0.00 ± 0.00	0.00 ± 0.00	19.89 ± 0.60	20.00 ± 0.71	19.78 ± 0.83
MRSA 2499	0.00 ± 0.00	0.00 ± 0.00	20.00 ± 1.12	19.44 ± 1.42	19.56 ± 1.42
MRSA 142	0.00 ± 0.00	0.00 ± 0.00	19.22 ± 0.67	19.33 ± 1.12	19.56 ± 0.73
MRSA 1429	0.00 ± 0.00	0.00 ± 0.00	18.44 ± 1.81	19.00 ± 1.00	18.67 ± 1.22
*E. coli* TISTR 887	0.00 ± 0.00	0.00 ± 0.00	0.00 ± 0.00	0.00 ± 0.00	0.00 ± 0.00
**LB/2+su**					
*S. aureus* TISTR 517	0.00 ± 0.00	0.00 ± 0.00	18.89 ± 1.45	19.33 ± 0.71^a^	19.78 ± 0.83
MRSA 1096	0.00 ± 0.00	0.00 ± 0.00	19.22 ± 1.09^a^	19.56 ± 1.24	19.44 ± 1.74
MRSA 2499	0.00 ± 0.00	0.00 ± 0.00	18.78 ± 1.20^a^	19.44 ± 1.01	19.56 ± 1.01
MRSA 142	0.00 ± 0.00	0.00 ± 0.00	18.44 ± 1.88^a^	18.67 ± 1.00^a^	18.89 ± 0.60^a^
MRSA 1429	0.00 ± 0.00	0.00 ± 0.00	17.00 ± 2.29^a^	18.44 ± 1.01	18.89 ± 0.93
*E. coli* TISTR 887	0.00 ± 0.00	0.00 ± 0.00	0.00 ± 0.00	0.00 ± 0.00	0.00 ± 0.00
**LB/2+lac**					
*S. aureus* TISTR 517	0.00 ± 0.00	0.00 ± 0.00	20.22 ± 1.09^b^	19.78 ± 1.09	19.89 ± 1.05
MRSA 1096	0.00 ± 0.00	0.00 ± 0.00	19.22 ± 0.83^a^	18.89 ± 0.60^a^	19.33 ± 0.71
MRSA 2499	0.00 ± 0.00	0.00 ± 0.00	19.00 ± 1.00^a^	18.22 ± 0.83^a^	19.00 ± 0.71
MRSA 142	0.00 ± 0.00	0.00 ± 0.00	18.56 ± 1.42^a^	18.78 ± 1.30	18.56 ± 1.13^a^
MRSA 1429	0.00 ± 0.00	0.00 ± 0.00	19.00 ± 0.87	19.22 ± 1.09	18.44 ± 1.01
*E. coli* TISTR 887	0.00 ± 0.00	0.00 ± 0.00	0.00 ± 0.00	0.00 ± 0.00	0.00 ± 0.00
**LB/2+glu**^a^					
*S. aureus* TISTR 517	0.00 ± 0.00	0.00 ± 0.00	0.00 ± 0.00^a^	0.00 ± 0.00^a^	0.00 ± 0.00^a^
MRSA 1096	0.00 ± 0.00	0.00 ± 0.00	0.00 ± 0.00^a^	0.00 ± 0.00^a^	0.00 ± 0.00^a^
MRSA 2499	0.00 ± 0.00	0.00 ± 0.00	0.00 ± 0.00^a^	0.00 ± 0.00^a^	0.00 ± 0.00^a^
MRSA 142	0.00 ± 0.00	0.00 ± 0.00	0.00 ± 0.00^a^	0.00 ± 0.00^a^	0.00 ± 0.00^a^
MRSA 1429	0.00 ± 0.00	0.00 ± 0.00	0.00 ± 0.00^a^	0.00 ± 0.00^a^	0.00 ± 0.00^a^
*E. coli* TISTR 887	0.00 ± 0.00	0.00 ± 0.00	0.00 ± 0.00^a^	0.00 ± 0.00^a^	0.00 ± 0.00^a^

a Significantly decreased antibacterial activity.

b Significantly increased antibacterial activity, NC= Negative control, LB/2 = Half-formula of Luria-Bertani broth, LB/2+su = Half-formula of Luria-Bertani broth supplemented with sucrose, LB/2+lac = Half-formula of Luria-Bertani broth supplemented with lactose, LB/2+glu = Half-formula of Luria-Bertani broth supplemented with glucose.

### 3.4 Effect of nitrogen sources

**Fig. 4:**
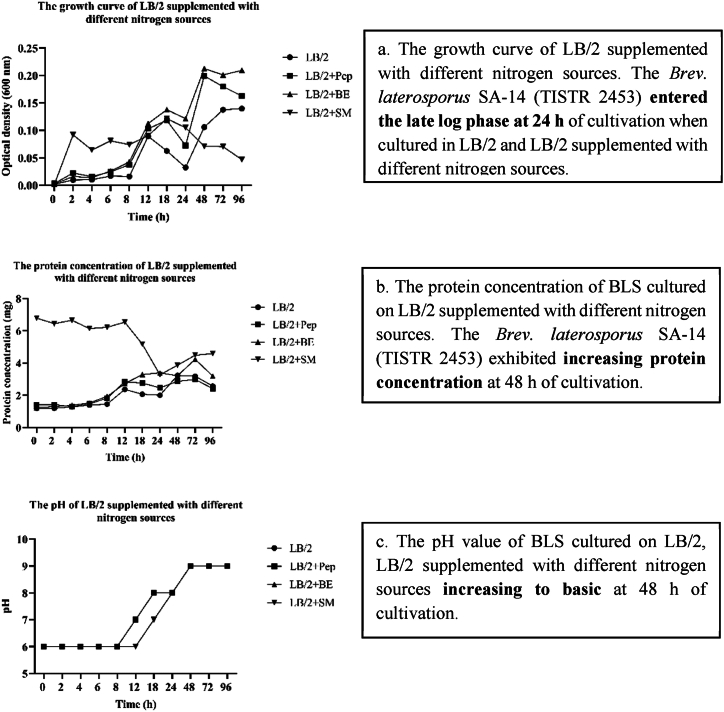
The bacteriocin-like substances (BLS) produced from SA-14 strain by culture on LB/2 supplemented with different nitrogen sources were improved, (a.) growth curve of *Brev. laterosporus* SA-14 (TISTR 2453), (b.) protein concentration of BLS in different nitrogen sources, (c.) pH value of BLS in different nitrogen sources.

Nitrogen is essential for the synthesis of key cellular components such as proteins and nucleic acids. Once these primary functions are fulfilled, nitrogen is utilized to produce secondary metabolites during the late log phase of bacterial growth [45]. Bacteria produce hydrolytic enzymes such as peptidases, which can digest proteins in the culture medium into amino acids or short peptides [46]. These components are integral to the cell membrane and cell wall and contribute to cell growth and increased cell mass [47]. In this study, the basal medium (LB/2) was supplemented with different nitrogen sources, and the results showed that LB/2 and LB/2 supplemented with different nitrogen sources entered the late log phase after 24 h of cultivation (Fig. 4 a). Significant antibacterial activity was observed for all bacterial indicators at p < 0.05 at 24 h of cultivation when LB/2 cells were supplemented with peptone and skim milk. In contrast, LB/2 and LB/2 supplemented with beef extract exhibited antibacterial activity after 48 h of cultivation (Table 2), corresponding to a decrease in the protein concentration after 24 h (Fig. 4 b) and an increase after 48 h of cultivation. The pH became basic after 48 h of cultivation in both LB/2 and LB/2 supplemented with different nitrogen sources (Fig. 4 c). Therefore, the SA-14 strain cultured on LB/2 supplemented with beef extract showed antibacterial activity at 48 h, similar to that of LB/2 medium without an added nitrogen source. This causes the beef extract to contain proteins and peptides that must be broken down into amino acids for growth, and it has been reported that 1.0 % beef extract increased antimicrobial substances produced by *Streptococcus lactis* subsp. *diacetylactis* S1-67/C [48]. In contrast, peptone consists of short peptides and amino acids that require less digestion time than beef extract and can be readily absorbed by bacteria. This results in faster bacterial growth and earlier entry into the late logarithmic phase. Skim milk was found to be the best nitrogen source for enhancing BLS production by the SA-14 strain, as it contains casein and lactose, which provide a rich carbon source for energy and a nitrogen source that promotes cell mass growth and BLS production corresponding to previous study that exhibited milk concentration was increased from 5 to 30 wt%, the population of non-lactose-fermenting strains increased by around 1 log at stationary growth phase [49].This leads to faster and more prolific bacterial multiplication compared with culture media supplemented with other nitrogen sources. Consistent with the observed protein levels, protein levels began to decrease after 24 h of cultivation because bacteria utilized it for growth and as a precursor for secondary metabolites. Protein levels began to increase again as the pH became basic, and the bacteria began to produce BLS. Therefore, LB/2 supplemented with skim milk is the optimal formula for improving BLS production by the SA-14 strain.

**Table 2:** Antibacterial activity of SA-14 strain compared to the basal medium (LB/2) with and without supplementation of different nitrogen sources.

Zone of inhibition (mm ± SD)						
Organisms	NC	0–18 h	24 h	48 h	72 h	96 h
**LB/2**						
*S. aureus* TISTR 517	0.00 ± 0.00	0.00 ± 0.00	0.00 ± 0.00	19.56 ± 1.13	19.89 ± 0.78	20.22 ± 0.44
MRSA 1096	0.00 ± 0.00	0.00 ± 0.00	0.00 ± 0.00	19.78 ± 0.67	20.67 ± 0.71	20.44 ± 0.53
MRSA 2499	0.00 ± 0.00	0.00 ± 0.00	0.00 ± 0.00	19.44 ± 1.13	19.44 ± 0.88	19.67 ± 0.87
MRSA 142	0.00 ± 0.00	0.00 ± 0.00	0.00 ± 0.00	18.67 ± 0.50	19.22 ± 0.67	19.67 ± 0.50
MRSA 1429	0.00 ± 0.00	0.00 ± 0.00	0.00 ± 0.00	19.00 ± 0.87	19.56 ± 0.53	20.00 ± 0.50
*E. coli* TISTR 887	0.00 ± 0.00	0.00 ± 0.00	0.00 ± 0.00	0.00 ± 0.00	0.00 ± 0.00	0.00 ± 0.00
**LB/2+pep**						
*S. aureus* TISTR 517	0.00 ± 0.00	0.00 ± 0.00	14.67 ± 1.94^b^	19.11 ± 1.05	20.67 ± 0.71	21.56 ± 0.88^b^
MRSA 1096	0.00 ± 0.00	0.00 ± 0.00	14.67 ± 1.41^b^	18.22 ± 0.67^a^	20.33 ± 0.71	21.22 ± 0.44^b^
MRSA 2499	0.00 ± 0.00	0.00 ± 0.00	15.22 ± 1.39^b^	19.22 ± 0.67	20.11 ± 0.33	20.78 ± 0.44^b^
MRSA 142	0.00 ± 0.00	0.00 ± 0.00	14.33 ± 1.23^b^	17.67 ± 0.71^a^	19.44 ± 0.88	20.44 ± 0.88^b^
MRSA 1429	0.00 ± 0.00	0.00 ± 0.00	14.67 ± 1.66^b^	18.44 ± 0.73	20.00 ± 0.71	20.22 ± 0.67
*E. coli* TISTR 887	0.00 ± 0.00	0.00 ± 0.00	0.00 ± 0.00	0.00 ± 0.00	0.00 ± 0.00	0.00 ± 0.00
**LB/2+be**						
*S. aureus* TISTR 517	0.00 ± 0.00	0.00 ± 0.00	0.00 ± 0.00	17.22 ± 1.30^a^	20.11 ± 1.05	20.78 ± 0.83
MRSA 1096	0.00 ± 0.00	0.00 ± 0.00	0.00 ± 0.00	14.44 ± 1.51^a^	20.22 ± 0.97	20.67 ± 0.87
MRSA 2499	0.00 ± 0.00	0.00 ± 0.00	0.00 ± 0.00	15.33 ± 1.00^a^	19.56 ± 1.88	20.33 ± 1.23
MRSA 142	0.00 ± 0.00	0.00 ± 0.00	0.00 ± 0.00	16.78 ± 1.30^a^	20.44 ± 1.51^b^	20.67 ± 1.41^b^
MRSA 1429	0.00 ± 0.00	0.00 ± 0.00	0.00 ± 0.00	17.22 ± 1.64^a^	19.89 ± 0.78	19.78 ± 1.09
*E. coli* TISTR 887	0.00 ± 0.00	0.00 ± 0.00	0.00 ± 0.00	0.00 ± 0.00	0.00 ± 0.00	0.00 ± 0.00
**LB/2+sm**^b^						
*S. aureus* TISTR 517	0.00 ± 0.00	0.00 ± 0.00	18.67 ± 0.82^b^	25.78 ± 1.79^b^	25.00 ± 1.50^b^	25.11 ± 2.52^b^
MRSA 1096	0.00 ± 0.00	0.00 ± 0.00	18.00 ± 0.71^b^	27.00 ± 1.00^b^	27.33 ± 1.00^b^	26.44 ± 1.59^b^
MRSA 2499	0.00 ± 0.00	0.00 ± 0.00	17.89 ± 0.93^b^	25.11 ± 0.78^b^	24.67 ± 1.00^b^	24.56 ± 1.67^b^
MRSA 142	0.00 ± 0.00	0.00 ± 0.00	18.00 ± 1.00^b^	25.89 ± 0.78^b^	25.56 ± 1.01^b^	25.33 ± 1.12^b^
MRSA 1429	0.00 ± 0.00	0.00 ± 0.00	17.67 ± 2.40^b^	27.44 ± 1.13^b^	26.33 ± 1.87^b^	26.11 ± 2.71^b^
*E. coli* TISTR 887	0.00 ± 0.00	0.00 ± 0.00	0.00 ± 0.00^b^	0.00 ± 0.00	0.00 ± 0.00	0.00 ± 0.00

a Significantly decreased antibacterial activity.

b Significantly increased antibacterial activity, NC= Negative control, LB/2 = Half-formula of Luria-Bertani broth, LB/2+pep = Half-formula of Luria-Bertani broth supplemented with peptone, LB/2+be = Half-formula of Luria-Bertani broth supplemented with beef extract, LB/2+sm = Half-formula of Luria-Bertani broth supplemented with skim milk.

### 3.5 Partial purification of BLS using 80 % ammonium sulfate precipitation

The protein concentration in cell-free supernatant (CFS) was 3.05 ± 0.56 mg protein/mL, and after partial purification, the protein amount increased to 6.97 ± 1.12 mg protein/mL, representing a percent yield of 38.09 % (Table 3) with a similarly %recovery yield in previous studies, it was found that precipitating CFS produced by the SA-14 strain with 50 % ammonium sulfate resulted in an 88 % recovery yield [13], while CFS produced by *Pediococcus pentosaceus* PB2 precipitated with 80 % ammonium sulfate yielded a 40 % recovery [50]. This increase in protein was achieved using high concentrations of ammonium sulfate (80 %), which caused the protein molecules in the CFS to lose their water solubility (salting-out) and precipitate [51]. This method is suitable for partially purifying BLS, a type of protein soluble in CFS, as it allows for the separation of proteins from other substances and increases the yield by accumulating protein sediment. The active substances produced by SA-14 were partially purified and used for further testing.

**Table 3:** The percent yield of CFS and BLS produced by the SA-14 strain.

Sample	Volume (ml)	Protein concentration (mg protein/ml)	Total protein (mg)	% yield
Cell free supernatant	3000	3.05 ± 0.56	9150	100.00
Bacteriocin-like substance	500	6.97 ± 1.12	3485	38.09

### 3.6 Antibacterial activity of CFS and BLS by using minimum inhibitory concentration (MIC) and minimum bactericidal concentration (MBC)

The antibacterial activity of the smallest amount of substances or drugs required to cure bacterial strains or AMR was determined by MIC and MBC, which are vital for effective treatment without the overuse of antibiotics. In this study, the CFS exhibited a MIC value of 0.76 ± 0.00 mg protein/mL against all GPB indicators, while the MBC value was 1.53 ± 0.00 mg protein/mL for *S. aureus* TISTR 517, MRSA 1096, and MRSA 2499. For MRSA 142 and MRSA 1429, the MBC value was 0.76 ± 0.00 mg protein/mL (Table 4). The BLS exhibited an MIC value of 0.87 ± 0.00 mg protein/mL for *S. aureus* TISTR 517, MRSA 1096, and MRSA 2499, while MRSA 142 and MRSA 1429 had an MIC value of 0.44 ± 0.00 mg protein/mL. The MBC value of BLS was 0.87 ± 0.00 mg protein/mL for all GPB indicator strains (Table 4). Both CFS and BLS displayed bactericidal properties and exhibited dose-dependent effects, with antibacterial activity directly varying with BLS concentration. The mode of action of BLS affects the bacterial cell wall [52]. BLS produced by the SA-14 strain can inhibit the growth of GPB, but not GNB, because of the more complex cell membrane structure of GNB [22]. The outer membrane of GNB contains lipopolysaccharides (LPS) [53], which act as a strong barrier, making it more difficult for bacteriocins to penetrate and reach the inner membrane or peptidoglycan layer of the cell wall. Consequently, bacteriocins are less effective against GNB. In contrast, BLS exhibited strong antibacterial activity GPB, especially *S. aureus* and MRSA strains, due to the thick peptidoglycan layer in the cell wall of GPB. BLS can easily penetrate this structure and bind to peptidoglycan or other cell wall components, causing leakage of intracellular contents and leading to bacterial cell death [22,54]. This is consistent with the results of a previous study showing that *Brev. laterosporus* TK3 produces secondary metabolites that inhibit the growth of *Listeria monocytogenes*, *S. aureus*, and *Clostridium perfringens* [55]. Moreover, some studies have reported that *Brev. laterosporus* strains BGSP7, BGSP9, and BGSP11 produce secondary metabolites that inhibit the growth of GNB, including *Klebsiella pneumoniae* Ni9 and *Pseudomonas aeruginosa* MMA83 and Gram-positive *S. aureus* ATCC25923 and *Listeria monocytogenes* ATCC19111 [8]. In this study, we found that BLS produced by the SA-14 strain was more effective against MRSA strains that produce a D-zone than against non-D-zone strains. The D-zone strains are bacteria-inducible macrolide lincosamide-streptogramin (iMLSB)-resistant, which shows that the bacteria are susceptible to clindamycin but resistant to erythromycin [56].

**Table 4:** Antibacterial activity of CFS and BLS by using MIC and MBC.

Organisms	Cell free supernatant (mg protein/ml)		Bacteriocin-like substance (mg protein/ml)	
	MIC	MBC	MIC	MBC
*S. aureus* TISTR 517	0.76 ± 0.00	1.53 ± 0.00	0.87 ± 0.00	0.87 ± 0.00
MRSA 1096	0.76 ± 0.00	0.76 ± 0.00	0.87 ± 0.00	0.87 ± 0.00
MRSA 2499	0.76 ± 0.00	0.76 ± 0.00	0.87 ± 0.00	0.87 ± 0.00
MRSA 142	0.76 ± 0.00	1.53 ± 0.00	0.44 ± 0.00	0.87 ± 0.00
MRSA 1429	0.76 ± 0.00	1.53 ± 0.00	0.44 ± 0.00	0.87 ± 0.00
*E. coli* TISTR 887	>3.05 ± 0.00	>3.05 ± 0.00	6.97 ± 0.00	>6.97 ± 0.00

∗MIC, minimum inhibitory concentration; MBC, minimum bactericidal concentration.

Furthermore, the exact mechanisms of BLS production by SA-14 strain against MRSA strains, both D-zone and non-D-zone producers, will be studied in detail to provide a clearer understanding.

### 3.7 Synergistic effects of BLS produced by the SA-14 strain and vancomycin against MRSA

AMR is caused by the use of unregulated chemicals as conventional drugs. Therefore, reducing drug use is a good strategy for controlling AMR. Therefore, the combination of a conventional chemical drug and a BLS is a key strategy for controlling AMR. This study focused on the combination of vancomycin and BLS produced by the SA-14 strain. The results exhibited that the MIC value of vancomycin is 2 μg and that of BLS is 0.44 mg protein/mL for all bacterial indicators (Table 5). Furthermore, when vancomycin was combined with BLS, the MIC values were reduced to 1 μg and 0.22 mg protein/mL, respectively, for all bacterial indicators. Moreover, the fractional inhibitory concentration index (FICI) was 1.00, indicating that vancomycin combined with BLS had an additive effect and that the combined effect of vancomycin and BLS was equal to the sum of their individual effects. This implies that both substances work together without enhancing or diminishing the effects of each other. The additive interaction between vancomycin and BLS suggested that each agent contributed to bacterial inhibition through distinct mechanisms. BLS targets the bacterial cell membrane by forming pores, causing leakage of vital substances [57], while vancomycin targets the bacterial cell wall by repressing cell wall synthesis, altering cell membrane permeability, and inhibiting ribonucleic acid synthesis [58]; these independent actions combine additively. This finding aligns with those of previous studies that demonstrated the synergistic activity of brevibacillin, brevibacillin V, brevibacillin I, and brevibacillin V2 produced by *Brev. laterosporus* DSM 25 with tested antibiotics against Gram-negative pathogens. Additionally, brevibacillin 2V showed the highest synergistic activity with amikacin against *Acinetobacter baumannii* [59]. These interactions are vital for optimizing antimicrobial therapy and managing resistance.

**Table 5:** Additive effects of BLS produced by the SA-14 strain and vancomycin against MRSA.

Organisms	MIC			FICI	Interpreted
	Vancomycin (μg)	BLS (mg protein/ml)	Vancomycin (μg) + BLS (mg protein/ml)		
MRSA 142	2.00	0.44	1.00 + 0.22	1.00	Additive
MRSA 1429	2.00	0.44	1.00 + 0.22	1.00	Additive

∗MIC, minimum inhibitory concentration; FICI, fractional inhibitory concentration index; BLS, bacteriocin-like substances.

### 3.8 Determination of the molecular weight of BLS produced by the SA-14 strain using SDS-PAGE assay

**Fig. 5:**
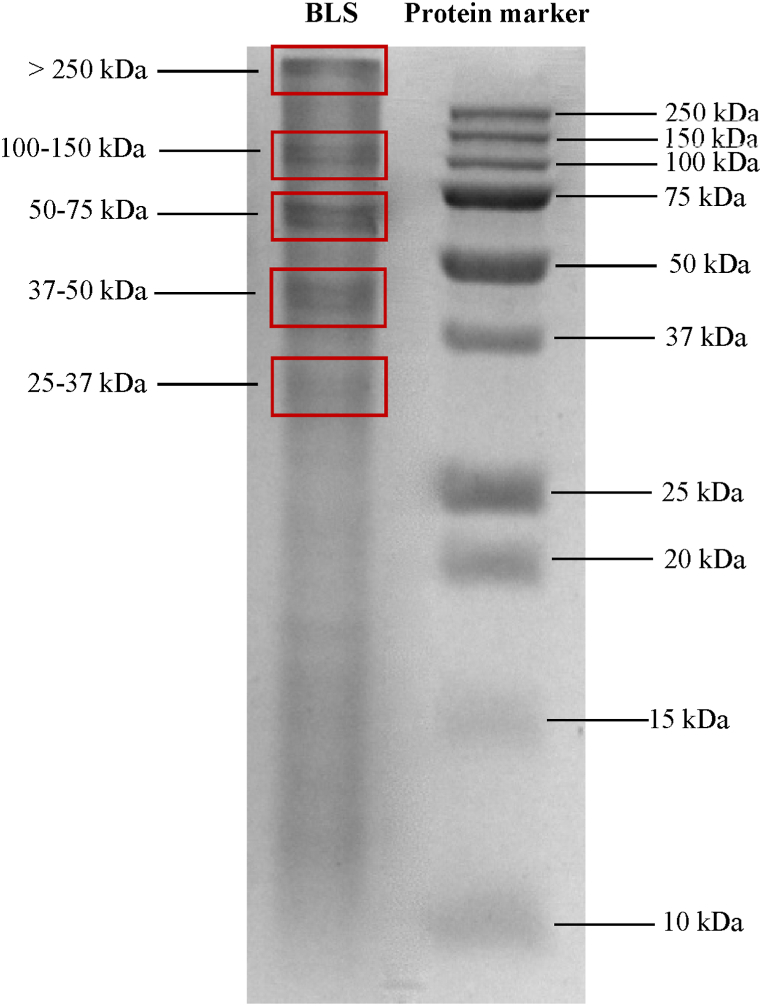
The SDS-PAGE assay of BLS shows five bands ranging from 25 kDa to over 250 kDa, as shown in Supplemental information. Full, unadjusted images are provided for reference.

Sodium Dodecyl Sulfate-Polyacrylamide Gel Electrophoresis (SDS-PAGE) is used to separate proteins based on their molecular weight [26]. When the partially purified protein BLS, with a concentration of 6.97 ± 1.12 mg protein/mL, produced by the SA-14 strain, was analyzed for its molecular weight, the results showed that BLS separated into five bands: >250 kDa, 100–150 kDa, 50–75 kDa, 37–50 kDa, and 25–37 kDa (Fig. 5). This is consistent with a previous study that showed that the anti-MRSA protein produced by the SA-14 strain after culture in LB broth for 24 h was concentrated with a concentrator tube cutoff of 3 kDa, resulting in a single band at 116 kDa [19]. In contrast, when the BLS from SA-14 strain was partially purified using Sp-Sepharose fast flow column chromatography, it showed a molecular weight of 6.9 kDa [13]. The difference in the molecular weight of proteins was due to the different purification methods used. In this study, 80 % ammonium sulfate was used to precipitate proteins. High concentrations of ammonium sulfate can lead to the formation of precipitated protein aggregates [51], which can result in a broad band on the SDS-PAGE gel. The antibacterial activity of BLS against each band was determined using an overlay assay.

### 3.9 In vitro cytotoxicity on HacaT cells

**Fig. 6:**
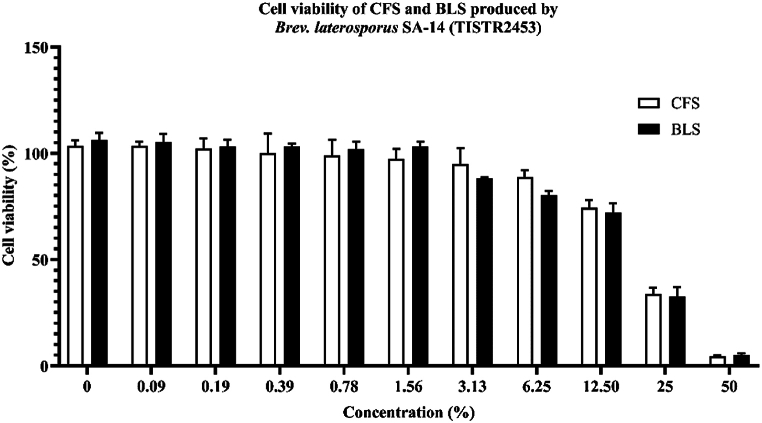
Cell viability of cell free supernatant (CFS) and bacteriocin-like substances (BLS) produced by SA-14 strain.

An MTT assay was used to evaluate the cytotoxicity of BLS in keratinocyte (HaCaT) cells. The results showed that both CFS and BLS were toxic to HaCaT cells, with a cell viability of less than 80 % at concentrations ranging from 50 to 12.5 %, corresponding to protein concentrations of 1.53–0.38 mg protein/mL for CFS and 3.49–0.87 mg protein/mL for BLS. However, cell viability remained above 80 % at a concentration of 6.25 % for both CFS and BLS, with protein concentrations of 0.19 mg protein/mL and 0.44 mg protein/mL, respectively (Fig. 6). The IC_50_ values of CFS and BLS were determined to be 24.32 % and 23.78 %, respectively. The cytotoxicity of BLS produced by the SA-14 strain is dose-dependent, consistent with previous studies that exhibited bacteriocin produced by *B. subtilis* GAS 101 at a concentration of 2.88 mg/mL resulted in more than 70 % cell viability in the Caco-2 cell line, whereas at 5.76 mg/mL, cell viability dropped below 20 % [28]. Additionally, crude extract substances produced by *B. subtilis* at a concentration of 4 % showed lower cytotoxicity compared to *B. subtilis* at a concentration of 8 % [60]. HaCaT cells, which are human epidermal keratinocytes, possess a lipid bilayer membrane structure that includes both hydrophilic and hydrophobic fatty acid tails [61]. The hydrophobic nature of BLS allowed it to penetrate and diffuse into the lipid bilayer. Moreover, the hydrophobicity of BLS leads to the accumulation of substances within target cells, causing toxicity or damage to cell membranes. This is supported by previous studies that demonstrated that the hydrophobicity of cyanobacterial peptide hepatotoxins such as microcystins (MC) markedly enhanced lactate dehydrogenase leakage, indicating plasma membrane damage [62]. Further studies are required to fully understand the mechanisms by which BLS exerts its toxic effects on human cell lines. This study could contribute to the development of BLS as a novel anti-MRSA agent in the future.

### 3.10 Wound healing activity of BLS produced by SA-14 strain

**Fig. 7:**
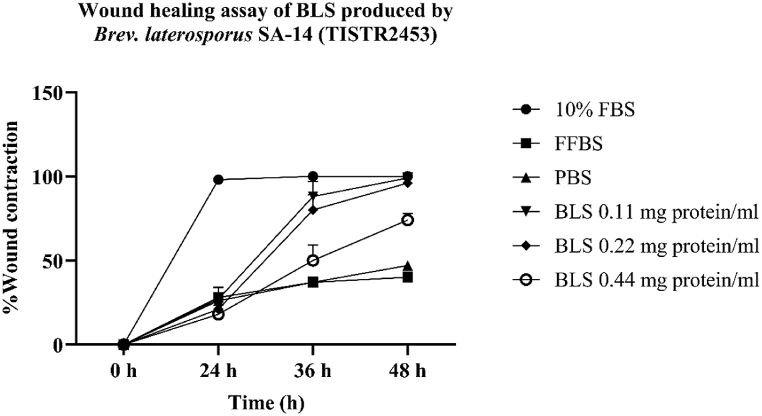
%Wound closure of BLS produced by SA-14 strain, FBS= Fetal bovine serum, FFBS=Free fetal bovine serum, PBS= Phosphate-buffered saline, BLS= Bacteriocin-like substance.

**Fig. 8:**
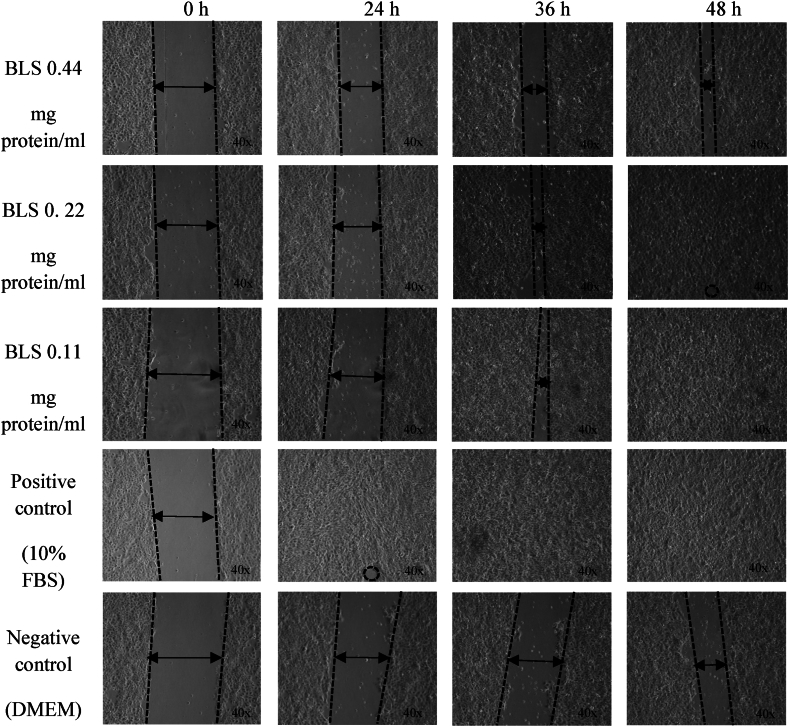
The wound closure of BLS produced by SA-14 strain at concentrations of 0.11, 0.22, and 0.44 mg protein/mL compared to positive control 10 % fetal bovine serum and negative control DMEM.

A wound healing assay was performed using a scratch assay. When cells or skin are damaged, they undergo four phases of healing: hemostasis, inflammatory response, proliferation, and dermal remodeling [63]. Several studies have reported five phases of wound healing: inflammation, wound contraction, collagen formation, epithelialization, and cicatrization [64]. Despite these differences, the overall wound-healing process was consistent. When a wound occurs, hemostasis is stimulated by resident macrophages (Langerhans cells). This is followed by an inflammatory response with the secretion of various inflammatory cytokines, including interleukins (IL-1, IL-6, IL-10, IL-17, IL-18, IL-22) and tumor necrosis factor-alpha (TNF-α) [63]. Subsequently, tissue remodeling begins with collagen formation, tissue proliferation, and maturation, leading to the completion of the healing process. This study focused on the wound-healing properties of BLS produced by the SA-14 strain in HaCaT cells. The results showed that BLS at concentrations of 3.13–1.50 % (0.22–0.11 mg protein/mL) achieved 96.00 ± 6.00 % to 99.00 ± 1.00 % wound closure after 48 h of incubation, which was comparable to the positive control, 10 % FBS, which showed 100 % wound closure in the same time frame. However, BLS at a concentration of 6.25 % (0.44 mg protein/mL) showed a lower wound closure percentage of 74.00 ± 4.00 % after 48 h (Fig. 7, Fig. 8). This is consistent with previous studies showing that crude extract produced by the SA-14 strain at 40 μg induced HaCaT cell migration and healing in the scratch assay [24,65]. Furthermore, crude extract concentrations of 10–80 μg did not exhibit cytotoxicity to white blood cells (WBCs) and even promoted WBC proliferation [24]. Additionally, Nisin A has been shown to significantly promote HaCaT cell migration by decreasing the levels of TNF-α, IL-6, and IL-8 [66]. Bacteriocin produced by *Lactobacillus rhamnosus* also induced complete wound healing in mice after 14 days of treatment [67]. Despite these findings, the mechanisms of wound healing induced by BLS produced by *Brevibacillus* spp., particularly *Brev. laterosporus* SA-14 (TISTR 2453), remain unclear and require further study to effectively apply these findings in medical practice in the future.

## 4 Conclusion

The global rise in AMR bacteria, particularly MRSA, poses a serious public health challenge because of their resistance to common antibiotics. Therefore, there is an urgent need for novel antimicrobial agents. Bacteriocin-like substances (BLS) produced by bacteria show promise for combating resistant strains and offer a viable solution to this issue, with potential industrial applications for increased yield. This study found that *Brevibacillus laterosporus* SA-14 (TISTR 2453) can produce substances that inhibit the growth of the GPB *S. aureus* and MRSA tested by the cross-streak method. Moreover, sugars as the carbon sources are not suitable for improving BLS production by the SA-14 strain, whereas nitrogen sources, particularly skim milk, significantly enhanced BLS production and antibacterial activity, especially against all MRSA strains. The SDS-PAGE analysis of BLS showed five protein bands with molecular weights of 25–250 kDa. The MIC of vancomycin and BLS was 1 μg and 0.44 mg protein/mL, respectively, for all bacterial indicators. Furthermore, when vancomycin was combined with BLS, the MIC values were reduced to 1 μg and 0.22 mg protein/mL, respectively, for all bacterial indicators. Moreover, the fractional inhibitory concentration index (FICI) was 1.00, which was interpreted as an additive for all MRSA strains. Additionally, at a concentration of 0.22 mg protein/mL, BLS did not show hemolytic activity, was non-cytotoxic to HaCaT cells, and induced complete wound healing in HaCaT cells after 48 h of treatment. Therefore, BLS produced by the SA-14 strain is suitable for controlling AMR, especially MRSA, and has the potential for topical application in wound dressings in the future.

## CRediT authorship contribution statement

**Husna Madoromae:** Writing – review & editing, Writing – original draft. **Apichart Atipairin:** Supervision, Methodology. **Malatee Tayeh:** Supervision, Methodology. **Monthon Lertcanawanichakul:** Writing – review & editing, Writing – original draft, Supervision, Methodology, Funding acquisition, Formal analysis, Conceptualization.

## Human ethics

This study does not involve human participants, and therefore, we did not submit an application for ethical approval.

## Ethics

This study was approved by the Institutional Biosafety Committee, Walailak University in a clearance no. WU-IBC-66-004.

## DNA deposition

The DNA sequences of strain SA14 in this study have been deposited in GenBank under the accession numbers KF718856.1 and is publicly accessible at

https://www.ncbi.nlm.nih.gov/genbank/ (https://www.ncbi.nlm.nih.gov/nuccore/KF718856.1).

## Data availability statements

No new data was generated for the research described in the article.

## Funding

This research was supported by 10.13039/501100010034Walailak University, Scholarships for High Potential Candidates to Enroll in Master Programs in Drug and Cosmetic Innovation (Contract no. 3/2022), 10.13039/501100010034Walailak University Graduated Scholarships (Contract no. 03/2565), and 10.13039/501100010034Walailak University Graduate Research Fund (Contact no. CGS-RF-2023/11).

## Declaration of competing interest

The authors declare that they have no known competing financial interests or personal relationships that could have appeared to influence the work reported in this paper.

## Figures and Tables

**figs1:**